# Polymorphisms in pattern recognition receptor genes of indigenous and White Leghorn breeds of chicken

**DOI:** 10.5194/aab-61-441-2018

**Published:** 2018-11-12

**Authors:** Santosh Haunshi, Arun Kumar Burramsetty, Kannaki Ramasamy, Rudra Nath Chatterjee

**Affiliations:** 1 ICAR-Directorate of Poultry Research, Rajendranagar, Hyderabad, India; 2 Current Address: MEXT Doctoral Scholar, Graduate School of Comprehensive Human Sciences, Department of Biomedical Sciences, University of Tsukuba, Tsukuba, Japan

## Abstract

Functional polymorphisms in pattern recognition receptors (PRRs) modulate innate immunity and play a crucial role in resistance or susceptibility to diseases. The present study was carried out to explore polymorphic patterns in the coding sequences of PRR genes *TLR3*, *TLR1LA* (TLRs), *MDA5*, *LGP2* (RLRs) and *NOD1* (NLR) in chicken breeds of India, namely *Ghagus* (GH), *Nicobari* (NB) and the exotic White Leghorn (WLH) breed. Out of 209 SNPs observed in five genes among three breeds, 117 were synonymous (Syn) and 92 were non-synonymous (NS) SNPs. In TLR genes the highest polymorphism was observed in NB (16, 28) compared to GH (14, 16) and WLH (13, 19) breeds. In the *MDA5* gene the highest polymorphism was observed in GH (12) compared to NB (eight) and WLH (four) breeds. However, an almost similar level of polymorphism was observed in the *LGP2* gene among the three breeds. In the *NOD1* gene, the highest polymorphism was observed in NB (27), followed by WLH (11) and GH (10) breeds. The overall highest number of SNPs was observed in NB (90), followed by GH (62) and the WLH (57) breed. With regard to variation in polymorphism among different classes of PRRs, the study revealed the highest polymorphism in TLRs compared to *NOD1* and the RLR class of PRRs. Further, the domain locations of various Syn and NS SNPs in each PRR among the three breeds were identified. In silico analysis of NS SNPs revealed that most of them had a neutral effect on protein function. However, two each in *TLR1LA* and *LGP2* and one in the *MDA5* gene were predicted to be deleterious to protein function. The present study unravelled extensive polymorphism in the coding sequences of the TLR and NLR class of PRR genes, and the polymorphism was higher in indigenous chicken breeds.

## 1 Introduction

Pattern recognition receptors (PRRs) are highly conserved germ-line-encoded molecules which recognize pathogen-associated molecular patterns (PAMPs) that are an integral part of pathogens. Recognition of PAMPs by PRRs leads to activation of innate immune response by inducing type I interferons and pro-inflammatory cytokines. Thus, PRRs work as first line of defence against invading pathogens. In chickens, three classes of PRRs, such as Toll-like receptors (TLRs), retinoic acid-inducible gene-I-like (RIG-I) receptors (RLRs), and nucleotide-binding and oligomerization-domain-like receptors (NLRs), have been described (Chen et al., 2013). The role of TLRs in the innate immune system and resistance to various infectious diseases has been well established (Keestra et al., 2013). A previous study reported the association of polymorphism in the *TLR4* gene with susceptibility to *Salmonella enterica* serovar Typhimurium infection in chickens (Leveque et al., 2003). Recently, the importance of other classes of PRRs such as RLR (Hayashi et al., 2014) and NLRs (Tao et al., 2015, 2017) in various infectious diseases was reported. Genetic variation in the coding sequences (CDSs) of PRRs, which results in changes in amino acid sequence, has the potential to affect the function of protein receptors and thereby modulate the resistance or susceptibility to infectious diseases (Jaeger et al., 2015). Therefore, SNPs in the CDSs of PRR genes may alter the activity of PRRs and thereby affect the resistance or susceptibility to infectious diseases. This is one of the mechanisms by which the genetic make-up of an organism can influence resistance and tolerance to infectious agents. Indigenous chicken breeds are known for their hardiness and ability to survive under adverse conditions with little or no healthcare. Natural selection over the years under resource-poor suboptimum conditions made indigenous chickens more robust and tolerant or resistant to various diseases, particularly of bacterial and parasitic origin. On the other hand, exotic breeds such as the White Leghorn have been subjected to genetic improvement for economic traits and may not be that hardy or resistant to diseases (Besbes, 2009). Therefore, the present study was carried out with the aim of exploring and comparing polymorphic patterns in PRR genes such as *TLR3*, *TLR1LA*, *MDA5*, *LGP2* and *NOD1* in chicken breeds of India (*Ghagus* and *Nicobari*) vis a vis the exotic White Leghorn breed.

**Table 1:** Number of SNPs in the PRR genes in three breeds of chicken.

	*TLR3*		*TLR1LA*		*MDA5*		*LGP2*		*NOD1*		Total	
Breed	Syn	NS	Syn	NS	Syn	NS	Syn	NS	Syn	NS	Syn	NS
WLH	6	7	11	8	1	3	7	3	5	6	30	27
*Ghagus*	7	7	8	8	8	4	8	2	4	6	35	27
*Nicobari*	6	10	16	12	6	2	7	4	17	10	52	38
Total	19	24	35	28	15	9	22	9	26	22	117	92

Syn: synonymous, NS: non-synonymous, SNPs: single nucleotide polymorphisms, PRR: pattern recognition receptors.

## 2 Materials and methods

### 2.1 Experimental birds

Three breeds of chicken, namely *Ghagus* (GH), *Nicobari* (NB) and White Leghorn (WLH), were used in this study. GH and NB are indigenous to India: GH is from mainland India (Karnataka), while NB is from the Nicobar Islands. Pedigreed random mating populations of these breeds are maintained at the institute farm of the ICAR Directorate of Poultry Research, Hyderabad. Representative birds of the same age (four males) from each breed were selected and sacrificed for collection of spleen samples. Samples were collected on ice and processed immediately for RNA isolation. The experiment was carried out with the approval of the Institutional Animal Ethics Committee (IAEC).

### 2.2 RNA extraction and cDNA preparation

Total RNA from spleen samples was extracted using TRIzol (Invitrogen, Carlsbad, CA) following the manufacturer's instructions. RNA thus obtained was quantified spectrophotometrically and converted to cDNA using a ProtoScript II First Strand cDNA conversion kit (New England Biolab Inc., MA, USA) following the manufacturer's instructions. Briefly, a reaction volume of 20 µL containing 1 µg of RNA, 5 µM oligo $d(T{)}_{23}$ VN primer, 10 µL of reaction mix (2×) and 2 µL of enzyme mix (10×) was incubated at 42 ${}^{\circ }$C for 1 h to convert RNA into cDNA, and the reaction was inactivated at 80 ${}^{\circ }$C for 5 min. The converted cDNA was confirmed by amplifying a 150 bp fragment of the β-actin gene, and positive samples were stored at -20 ${}^{\circ }$C until further use as a template for PCR reactions.

**Table 2:** Polymorphisms in the *TLR3* gene of different chicken breeds.

Nucleotide	Amino acid	Reference	Polymorphic	Type	Location	Breed
position (bp)	position	codon (AA)	codon (AA)	of SNPs	of SNPs	
50	17	TTT (F)	TCT (S)	NS	UR	WLH, NB
203	68	GAT (D)	GTT (V)	NS	LRR	NB, GH
274	92	AAA (K)	CAA (Q)	NS	LRR	NB, GH
293	98	AAT (N)	AGT (S)	NS	LRR	NB
522	174	CGT (R)	CGC (R)	Syn	UR	GH
657	219	GCG (A)	GCA (A)	Syn	UR	WLH, NB, GH
732	244	GAG (E)	GAA (E)	Syn	LRR	WLH, NB, GH
948	316	CAT (H)	CAA (Q)	NS	LRR	NB
949, 950	317	TTA (L)	AAA (K)	NS	LRR	NB
999	333	CTT (L)	CTG (L)	Syn	LRR	NB, GH
1164	388	ACT (T)	ACC (T)	Syn	LRR	WLH, NB, GH
1197	399	AGG (R)	AGC (S)	NS	LRR	WLH, NB, GH
1247	416	GAG (E)	GGG (G)	NS	UR	WLH, NB, GH
1538	513	AGG (R)	AAG (K)	NS	LRR	WLH, NB, GH
1781	594	GCG (A)	GTG (V)	NS	LRR_CT	WLH, NB
2013	671	CCG (P)	CCA (P)	Syn	UR	WLH
2108	703	GCT (A)	GTT (V)	NS	TIR	WLH, GH
2299	767	ACT (T)	TCT (S)	NS	TIR	WLH, GH
2481	827	GCT (A)	GCA (A)	Syn	TIR	NB
2499	833	GAG (E)	GAA (E)	Syn	TIR	WLH, NB, GH
2619	873	GTC (V)	GTT (V)	Syn	TIR	WLH, GH

LRR: leucine-rich repeat domain, LRR_CT: leucine-rich repeat C-terminal domain, TIR: the Toll–interleukin-1 receptor homology domain, NS: non-synonymous, Syn: synonymous, UR: unknown region, GH: *Ghagus*, NB: *Nicobari*, WLH: White Leghorn.

### 2.3 Design of primers

Primer pairs for amplifying and sequencing the coding regions of PRR genes (*TLR3*, *TLR1LA*, *LGP2*, *MDA5* and *NOD1*) were designed using the online primer designing tool Primer3Plus (http://www.bioinformatics.nl/primer3plus, last access: 12 March 2016) with sequences retrieved from the UCSC genome browser. Primers were checked for their specificity with Primer-BLAST (https://www.ncbi.nlm.nih.gov/tools/primer-blast, last access: 4 May 2016) and used for PCR amplification and sequencing (Table S1 in the Supplement).

### 2.4 PCR amplification and sequencing

Fragments of coding regions were amplified through PCR. The reaction was carried out in a 20 µL volume containing 4 µL of 5× high-fidelity buffer, 0.5 U of KAPA HiFi HotStart DNA polymerase (Kapa Biosystems, Cape Town, South Africa), 400 µM dNTPs, 0.2 µM each of forward and reverse primers, and 2 µL of cDNA template. PCR amplification was carried out using initial denaturation at 95 ${}^{\circ }$C for 5 min, followed by 40 cycles at 98 ${}^{\circ }$C for 20 s, annealing at *Ta* (Table S1) for 30 s and extension at 72 ${}^{\circ }$C for 40 s, with a final extension at 72 ${}^{\circ }$C for 5 min. Amplified products were separated on agarose gel and desired fragments were gel eluted using a QIAquick gel extraction kit (Qiagen GmbH, Hilden, Germany) following the manufacturer's instructions. These eluted products were sequenced bidirectionally using BigDye terminator sequencing on an ABI 3730 sequencer (Bioserve Biotehnologies Pvt. Ltd., Hyderabad, India). A stepwise direct sequencing strategy was chosen using primer pairs designed to produce overlapping fragments. This way it was possible to obtain an unambiguous sequence covering the entire coding sequence of PRR genes.

The coding sequences of all five genes from the three breeds were deposited in GenBank. The accession numbers are as follows. *TLR3*: MF576160 (WLH), MF576161 (NB) and MF576162 (GH); *TLR1LA*: MF563596 (WLH), MF563597 (NB) and MF563598 (GH); *MDA5*: MF563590 (GH), MF563591 (WLH) and MF563592 (NB); *LGP2*: MF563593 (WLH), MF563594 (NB) and MF563595 (GH); *NOD1*: MF576163 (WLH), MF576164 (NB) and MF576165 (GH).

**Table 3:** Polymorphisms in the *TLR1LA* gene of different chicken breeds.

Nucleotide	Amino acid	Reference	Polymorphic	Type	Location	Breed
position (bp)	position	codon (AA)	codon (AA)	of SNPs	of SNPs	
432		TTA (L)	TTG (L)	Syn	Unknown	NB
733	245	ACA (T)	GCA (A)	NS	LC R	NB
746	249	ACG (T)	ATG (M)	NS	LC R	NB (Del.)
860	288	GTG (V)	GAG (E)	NS	UR	WLH, NB, GH
1076	359	CGA (R)	CCA (P)	NS	UR	WLH, NB
1122	374	GAC (D)	GAT (D)	Syn	UR	NB
1162	388	ATA (I)	TTA (L)	NS	LRR	NB
1191	397	GAG (E)	GAA (E)	Syn	LRR	WLH, NB3
1293	431	TGC (C)	TGT (C)	Syn	LRR	NB, GH
1312	438	GCA (A)	TCA (S)	NS	UR	WLH, NB
1312, 1313	438	GCA (A)	AAA (K)	NS	UR	NB (two sites)
1412	471	AAT (N)	AGT (S)	NS	UR	WLH, NB, GH
1471	491	CGG (R)	AGG (R)	Syn	LRR	NB, GH
1557	519	GCT (A)	GCC (A)	Syn	UR	WLH, NB, GH
1623	541	TCG (S)	TCA (S)	Syn	LRR_CT	WLH, NB, GH
1728	576	ACG (T)	ACA (T)	Syn	LRR_CT	WLH, GH
1770	590	ACG (T)	ACT (T)	Syn	UR	WLH, NB
1770	590	ACG (T)	ACA (T)	Syn	UR	GH
1832	611	CCG (P)	CTG (L)	NS	TM	WLH, NB, GH
1890	630	ACG (T)	ACA (T)	Syn	UR	WLH, NB
2070	690	AAA (K)	AAG (K)	Syn	TIR	NB
2133	711	TCA (S)	TCG (S)	Syn	TIR	WLH, NB, GH
2133	711	TCA (S)	TCC (S)	Syn	TIR	WLH, NB, GH
2189	730	AAG (K)	AGG (R)	NS	TIR	WLH, NB, GH
2205	735	AAC (N)	AAT (N)	Syn	TIR	WLH, NB, GH
2238	746	CCG (P)	CCA (P)	Syn	TIR	WLH, NB,
2245	749	CCA (P)	TCA (S)	NS	TIR	WLH, NB, GH
2275	759	CTG (L)	TTG (L)	Syn	TIR	NB
2301	767	ACG (T)	ACC (T)	Syn	TIR	WLH, NB
2363	788	GCT (A)	GTT (V)	NS	TIR	GH (Del.)
2364	788	GCT (A)	GCC (A)	Syn	TIR	WLH
2370	790	AAC (N)	AAT (N)	Syn	TIR	WLH, NB
2381	794	CCG (P)	CTG (L)	NS	UR	GH
2443	815	TGT (C)	CGT (R)	NS	UR	WLH, NB, GH
2453	818	AAG (K)	ACG (T)	NS	UR	NB

LRR: leucine-rich repeats, LRR_CT: leucine-rich repeats C-terminal domain NS: non-synonymous, Syn: synonymous, UR: unknown region, GH: *Ghagus*, NB: *Nicobari*, WLH: White Leghorn, Del.: deleterious, TIR: the Toll–interleukin-1 receptor homology domain.

### 2.5 Sequence analysis

Sequences in the forward and reverse directions of each fragment were aligned, checked for misread bases and edited using the CodonCode aligner v5.0.1 (CodonCode Corporation, USA). Thus, consensus sequences were multiple aligned with the ClustalW function in MEGA7 (https://www.megasoftware.net, last access: 11 April 2017) against respective reference sequences of red junglefowl (*Gallus gallus*). Synonymous (Syn) and non-synonymous (NS) SNPs were recorded and analysed for their effect (neutral or deleterious) of amino acid substitution on protein function using an online tool, the protein variation effect analyser (https://provean.jcvi.org/index.php, last access: 5 May 2017). Further, a simple modular architecture research tool (SMART) was used to identify the locations of SNPs on different domains of PRRs (Letunic et al., 2012).

## 3 Results and discussion

Coding sequences of five genes belonging to three different classes of PRRs (TLRs, RLRs and NLRs) were studied for functional polymorphisms in GH, NB and WLH breeds. A total of 209 SNPs in five genes were observed among the three breeds: of these, 117 were Syn and 92 were NS SNPs. The highest polymorphism was observed in TLRs compared to *NOD1* and the RLR class of PRRs. NS SNP results in the substitution of amino acid, and that leads to changes in the functions of receptors which can be advantageous, neutral or deleterious to the functions of proteins, while variation due to Syn substitution is expected to be neutral (Downing, et al., 2010). Amino acid substitutions due to polymorphism in the leucine-rich repeats (LRR) domain of PRRs may affect the ability of receptors to recognize PAMPs, while those in the transmembrane domain may lead to defects in intracellular receptor transport. Finally, those in the internal domain may result in altered interaction with adaptor proteins or disrupted dimerization (Kutikhin et al., 2013). Gene-wise SNPs in the CDSs of various PRR genes are described in the following sections.

**Table 4:** Polymorphisms in the *MDA5* gene of different chicken breeds.

Nucleotide	Amino acid	Reference	Polymorphic	Type	Location	Breed
position (bp)	position	codon (AA)	codon (AA)	of SNPs	of SNPs	
306	102	CCT (P)	CCC (P)	Syn	UR	NB, GH
422, 423	141	TTC (F)	TCT (S)	NS	UR	WLH
423	141	TTC (F)	TTT (F)	Syn	UR	NB, GH
428	143	GAG (E)	GGG (G)	NS	UR	GH
431	144	GAG (E)	GGG (G)	NS	UR	GH
434	145	GAC (D)	GGC (G)	NS	UR	GH (Del)
806	269	AGT (S)	AAT (N)	NS	UR	NB
816	272	AAC (N)	AAT (N)	Syn	UR	NB
1056	352	GTT (V)	GTG (V)	Syn	DEXDc	NB
1080	360	CCG (P)	CCA (P)	Syn	DEXDc	GH
1127	376	CAC (H)	CGC (R)	NS	DEXDc	GH, WLH
1169	390	AAA (K)	AGA (R)	NS	DEXDc	WLH
1218	406	ACA (T)	ACC (T)	Syn	DEXDc	GH
1551	517	GCA (A)	GCC (A)	Syn	DEXDc	GH
2064	688	ACA (T)	ACG (T)	Syn	UR	GH
2346	782	GGT (G)	GGC (G)	Syn	HELICc	WLH
2362	788	ATT (I)	GTT (V)	NS	HELICc	NB
2760	920	ACA (T)	ACG (T)	Syn	UR	NB, GH
2793	931	AAC (N)	AAT (N)	Syn	UR	GH
2802	934	ATT (I)	ATC (I)	Syn	UR	NB

DEXDc: DEAD-like helicase superfamily, HELICc: helicase superfamily C-terminal domain, NS: non-synonymous, Syn: synonymous, UR: unknown region, TM: transmembrane, GH: *Ghagus*, NB: *Nicobari*, WLH: White Leghorn, Del: deleterious.

### 3.1 *TLR3*

*TLR3* is an intracellular type I transmembrane receptor which recognizes the dsRNA of pathogens (viruses) and is known for its role in antiviral innate immunity in chickens. In this gene (CDS: 2691 bp) the highest number of NS SNPs was observed in NB compared to GH and WLH breeds (Table 1). A total of 10 NS SNPs were present in the extracellular domain, of which 8 were in the LRR domain, 1 was in the LRR_CT domain and 1 was in the unknown region, while only 2 NS SNPs were present in the intracellular TIR domain. Relatively higher polymorphism was observed in NB compared to the other two breeds. Polymorphism was higher at the LRR domain of *TLR3* of NB, as 8 out of 10 NS SNPs observed were present in the extracellular LRR domain. Four out of 10 NS SNPs (A293G, T948A, T949A and T950A) were exclusive to NB and two NS SNPs (A203T and A274C) were shared between the NB and GH breeds, while no NS SNP was found to be exclusive to the WLH breed (Table 2). All these NS SNPs located in the LRR domain of *TLR3* might play a role in the modulation of innate immunity since PAMPs are known to bind to the LRR domain of *TLR3*. Polymorphism in the coding sequence of the *TLR3* gene was also reported previously in different chicken breeds (Ruan et al., 2015). Similar to the present findings, higher NS SNPs were reported in Chinese native chickens compared to the WLH breed. At least 10 NS SNPs (T50C, A203T, A274C, A293G, T999G, G1197C, G1538A, C1781T, C2108T and A2299T) were shared between chicken breeds in this study and Chinese breeds (Huang et al., 2012). Various studies have reported the relationship between polymorphisms in the *TLR3* gene and resistance or susceptibility to different viral diseases in human beings (Kindberg et al., 2011; Sironi et al., 2012; Svensson et al., 2012; Lee et al., 2013; Studzińska et al., 2017). Similar relationships between polymorphisms in the *TLR3* gene and resistance or susceptibility to various diseases could exist in chickens as well.

### 3.2 *TLR1LA*

The *TLR1LA* gene located on chromosome 4 of chickens is involved in innate immunity against bacterial infections. The *TLR1LA* receptor recognizes triacylated and diacylated lipopeptides as PAMPs through the leucine-rich repeat domain (Keestra et al., 2013). In this gene (CDS: 2188 bp partial) the highest number of NS SNPs was also seen in NB compared to GH and WLH breeds (Table 1). Out of 12 NS SNPs observed in NB, 6 (A733G, C746T, A1162T, G1312A, C1313A and A2453C) were exclusive to this breed, while only 2 (C2363T and C2381T) out of 8 NS SNPs observed were exclusive to the GH breed. No NS SNP was found to be exclusive to the WLH breed (Table 3). Five out of six exclusive NS SNPs of NB are located in the LRR or LCR domain of *TRLA1LA* where PAMPs are known to bind, and hence these NS SNPs might play a key role in innate immunity. Further, substitution of amino acids in the TIR domain (K730R, P749S and A788V) of *TLR1LA* may influence signal transduction as TIR is the functional domain for the subsequent recruitment of intracellular adapter proteins (Werling et al., 2009). Functional polymorphisms in human *TLR1* and *TLR2* genes were reported to be associated with resistance or susceptibility to various bacterial and fungal diseases (Rosentul et al., 2014; Jaeger et al., 2015). Extensive polymorphism in the *TLR1LA* gene was also reported in Chinese native and commercial chicken breeds (Ruan and Zheng, 2011), and at least 13 SNPs (Syn and NS) were shared between breeds in the present study and those studied by Ruan and Zheng (2011). Out of eight NS substitutions located in the extracellular domain of *TLR1LA* (Table 3), four substitutions, namely T245A, T249M, R359P and N471S, were common to indigenous chicken breeds of India and China. Six NS substitutions were located in the intracellular domain of *TLR1LA*, of which two NS substitutions (K730R and P749S) were shared with Chinese native breeds. One polymorphic site (P611L) located in the transmembrane domain among the three breeds was also reported in Chinese breeds (Ruan and Zheng, 2011).

**Table 5:** Polymorphisms in the *LGP2* gene of different chicken breeds.

Nucleotide	Amino acid	Reference	Polymorphic	Type	Location	Breed
position (bp)	position	codon (AA)	codon (AA)	of SNPs	of SNPs	
42	14	GCG (A)	GCC (A)	Syn	DEXDc	NB
44	15	CTG (L)	CCG (P)	NS	DEXDc	NB (Del)
219	73	GAC (D)	GAT (D)	Syn	DEXDc	WLH, NB, GH
231	77	GTA (V)	GTG (V)	Syn	DEXDc	WLH, NB, GH
516	172	ACC (T)	ACT (T)	Syn	DEXDc	WLH, NB, GH
534	178	TTT (F)	TTC (F)	Syn	DEXDc	GH
575	192	TTG (L)	TGG (W)	NS	DEXDc	GH (Del)
634	212	CCC (P)	TCC (S)	NS	UR	WLH, NB
724	242	GAG (E)	AAG (K)	NS	UR	WLH, NB
741	247	CCC (P)	CCT (P)	Syn	UR	NB, GH
1176	392	TGC (C)	TGT (C)	Syn	HELICc	WLH, NB, GH
1194	398	GCG (A)	GCA (A)	Syn	UR	GH
1543	515	AAG (K)	GAG (E)	NS	UR	WLH, NB, GH
1635	545	CAT (H)	CAC (H)	Syn	UR	WLH, NB, GH
1881	627	ACG (T)	ACA (T)	Syn	UR	WLH
1980	660	TTC (F)	TTT (F)	Syn	UR	WLH

DEXDc: DEAD-like helicase superfamily, HELICc: helicase superfamily C-terminal domain, NS: non-synonymous, Syn: synonymous, UR: unknown region, GH: *Ghagus*, NB: *Nicobari*, WLH: White Leghorn, Del.: deleterious.

### 3.3 *MDA5*

The *MDA5* gene, also known as *IFIH1* (CDS: 3006 bp), located on chromosome 7 codes for melanoma-differentiation-associated protein 5 (*MDA5*) and is an important member of the RLR class of PRRs. In this gene the highest number of NS SNPs was observed in GH, followed by the WLH and NB breed (Table 1). Most of the NS SNPs (five) were located in an unknown region, while two were located in DEXDc (GH and WLH) and one in the HELICc domain (NB) of the *MDA5* receptor (Table 4). Out of four NS SNPs observed in GH, three (A428G, A431G and A434G) were exclusive to this breed; two NS SNPs (G806A and A2362G) are exclusive to NB and two NS SNPs (T422C and A1169G) are exclusive to WLH. Since most of the NS SNPs were located in an unknown region (domain) of the *MDA5* receptor, their possible role in innate immunity could not be delineated (Table 4). *MDA5* is one of the cytosolic PRRs that recognizes dsRNA and poly (I : C), a synthetic analogue. *MDA5* was reported to be involved in the induction of chicken IFN β production against avian influenza virus infection (Hayashi et al., 2014). Further, it was reported that *MDA5* recognizes the virus responsible for infectious bursal disease, and that leads to the activation of *MDA5*-related innate immunity and the upregulation of MHC class I (Lee et al., 2014). Polymorphism in the *IFIH1*–*MDA5* gene was linked to spontaneous clearance of the hepatitis C virus infection in humans (Hoffmann et al., 2015). Further, a link indicating polymorphism in the *MDA5* gene as a risk factor for the enterovirus 71 infection in humans was reported (Pang et al., 2014). However, the influence of polymorphism in the *MDA5* gene on resistance or susceptibility to various viral diseases in chickens has yet to be reported.

**Table 6:** Polymorphisms in the *NOD1* gene of different chicken breeds.

Nucleotide	Amino acid	Reference	Polymorphic	Type	Location	Breed
position (bp)	position	codon (AA)	codon (AA)	of SNPs	of SNPs	
50	17	CTG (L)	CAG (Q)	NS	UR	GH
52	18	ACA (T)	GCA (A)	NS	UR	NB
156	52	AAC (N)	AAT (N)	Syn	UR	NB
343	115	CAG (Q)	AAG (K)	NS	UR	NB
351	117	TCA (S)	TCG (S)	Syn	UR	NB
369	123	AAA (K)	AAG (K)	Syn	UR	NB
375	125	GTC (V)	GTT (V)	Syn	UR	WLH, NB, GH
378	126	GTG (V)	GTA (V)	Syn	UR	NB
408	136	CAA (Q)	CAG (Q)	Syn	UR	NB
459	153	GCG (A)	GCA (A)	Syn	UR	NB
542	181	GAA (E)	GGA (G)	NS	UR	GH
621	207	GGC (G)	GGT (G)	Syn	UR	NB
759	253	GTA (V)	GTG (V)	Syn	UR	WLH, NB
811	271	ACA (T)	TCA (S)	NS	UR	WLH
811, 813	271	ACA (T)	TCG (S)	NS	UR	NB
816	272	GAG (E)	GAA (E)	Syn	UR	NB
955	319	TTG (L)	CTG (L)	Syn	LCR	WLH, NB
993	331	TCT (S)	TCC (S)	Syn	LCR	WLH, NB, GH
1273	425	CTG (L)	TTG (L)	Syn	UR	NB
1346	449	AAC (N)	ACC (T)	NS	UR	GH
1414	472	TAC (Y)	CAC (H)	NS	UR	NB, WLH
1528	510	CGC (R)	TGC (C)	NS	UR	GH
1528	510	CGC (R)	GGC (G)	NS	UR	NB
1578	526	TCC (S)	TCT (S)	Syn	UR	WLH, NB, GH
1653	551	TGC (C)	TGT (C)	Syn	UR	NB
1688	563	CGT (R)	CAT (H)	NS	UR	NB, WLH
1740	580	AAC (N)	AAT (N)	Syn	UR	WLH, NB
1856	619	ACC (T)	ATC (I)	NS	UR	WLH, NB, GH
2119	707	GAT (D)	AAT (N)	NS	LCR	WLH
2231	744	AAG (K)	AGG (R)	NS	LRR	WLH, NB, GH
2240	747	TAT (Y)	TGT (C)	NS	LRR	NB
2389	797	GAA (E)	AAA (K)	NS	LRR	NB
2565	855	GGC (G)	GGA (G)	Syn	LRR	NB
2595	865	AAC (N)	AAT (N)	Syn	LRR	GH

LCR: low-complexity region, LRR: leucine-rich repeat domain, NS: non-synonymous, Syn: synonymous, UR: unknown region, GH: *Ghagus*, NB: *Nicobari*, WLH: White Leghorn.

### 3.4 *LGP2*

The laboratory of genetics and physiology 2 (*LGP2*–*DHX58*) gene (CDS: 2025 bp) is another cytosolic PRR belonging to the RLR class. *LGP2*, located on chromosome 27, encodes for the cytoplasmic DEx(D/H)-box helicase receptor that recognizes both ssRNA and dsRNA of virus origin (Zhu et al., 2014). An almost similar level of polymorphism was observed among the three breeds in the coding sequence of this gene. Only two NS SNPs were located on the DEXDc domain of the *LGP2* receptor, while the remaining three were located on an unknown region (domain) of the *LGP2* receptor (Table 5). One NS SNP (T44C) in *Nicobari* and one NS SNP (T575G) in GH were exclusive to these breeds and were located on the DEXDc domain of the *LGP2* gene (Table 5). *LGP2* is considered to be essential for the augmentation of *MDA5*-dependent signaling (Zhu et al., 2014) since it functions as a positive regulator of antiviral responses (Satoh et al., 2010). It is also reported to compensate for the lack of RIG-I PRR in chickens (Uchikawa et al., 2016). Polymorphism in the *LGP2* gene in grass carp (*Ctenopharyngodon idella*) was reported to be associated with resistance or susceptibility to grass carp reovirus infection (Wan et al., 2013).

### 3.5 *NOD1*

The nucleotide-binding oligomerization-domain-containing protein 1 (NOD1) gene, located on chromosome 2, codes (CDS: 2856 bp) for an NLR-type intracellular PRR. This PRR is comprised of a caspase recruitment domain at the N terminus, seven leucine-rich repeat regions at the C terminus, and one NACHT domain between the N and C termini (Tao et al., 2015). In the *NOD1* gene the highest number of NS SNPs was observed in NB compared to GH and WLH breeds (Table 1). Most of the NS SNPs were located in the unknown region of the protein, while three NS SNPs were present in the LRR domain and one in low-complexity region of the receptor (Table 6). Six NS SNPs (A52G, C343A, A811T, A813G, C1528G, A2240G and G2389A) were exclusive to NB and four NS SNPs (T50A, A542G, A1346C and C1528T) were exclusive to GH, while only two NS SNPs (A271T and G707A) were exclusive to WLH (Table 6). All exclusive SNPs of GH and the majority of exclusive SNPs of NB were situated in an unknown region (domain) of the *NOD1*, while two SNPs (A2240G and G2389A) of NB were located in the LRR domain of *NOD1* that constitutes the recognition motif for PAMPs. All these NS SNPs might play a role in innate immunity as they can alter the structure or function of receptors by virtue of change in amino acid composition and sequence. *NOD1* initiates innate immunity by recognition of a specific bacterial molecule: peptidoglycan as a ligand. Subsequent to ligand binding, the *NOD1* receptor undergoes conformation changes and self-oligomerization and induces nuclear factor κB (NF κB) activation independent of TLRs, resulting in the production of pro-inflammatory cytokines, chemokines and antimicrobial peptides (Shaw et al., 2008). Expression of the *NOD1* gene is found to be induced in *Salmonella enterica* serovar Enteritidis (Tao et al., 2015) and *Salmonella pullorum* (Tao et al., 2017) infections in chickens (Tao et al., 2015). Polymorphism in the *NOD1* gene in humans was reported to be linked with *Helicobacter pylori* pathogenesis (Hofner et al., 2007; Kara et al., 2010), *Chlamydia trachomatis* infection (Branković et al., 2015) and cytomegalovirus control (Fan et al., 2016). The NS SNPs observed in this study could play a role in resistance to various infectious diseases in chickens, and their effect on innate immunity needs to be investigated in detail through pathogen challenge studies.

## 4 Conclusions

The study unravelled extensive single nucleotide polymorphisms in the coding sequences of PRR genes, and the polymorphism was higher in TLRs and *NOD1* genes compared to RLR genes. With regards to polymorphism in the three breeds, the highest polymorphism was observed in indigenous chicken NB compared to GH and WLH breeds. The effects of functional polymorphisms in PRRs on resistance or susceptibility to various infectious diseases have been well established in humans and other domestic animals. Therefore, we speculate that polymorphisms in the CDSs of PRR genes could influence the resistance or susceptibility to various infectious diseases in chickens as well. To the best of our knowledge, few reports exist in the literature about the association of polymorphisms in PRR genes with disease resistance or susceptibility in chickens. Therefore, further studies are needed to examine the functional effects of these NS SNPs in PRR genes on resistance or susceptibility to various diseases in chickens. In silico analysis of NS SNPs for their possible effect on protein function revealed that most of them had a neutral effect on protein function. However, five SNPs (two each in *TLR1LA* and *LGP2* and one in the *MDA5* gene) were predicted to be deleterious in nature. These deleterious substitutions are of greater interest as they can affect the structure or function of the protein receptor.

## Supplement

10.5194/aab-61-441-2018-supplement The supplement related to this article is available online at: https://doi.org/10.5194/aab-61-441-2018-supplement.

## Data Availability

The data from the paper are available upon request from the corresponding author.
