# Supplementary material for: Polymorphisms in pattern recognition receptor genes of indigenous and White Leghorn breeds of chicken
Source: Arch Anim Breed. 2018 Nov 12;61(4):441–9. doi: 10.5194/aab-61-441-2018 (PMC7065405; doi:10.5194/aab-61-441-2018)
Supplement: The supplement related to this article is available online at: https://doi.org/10.5194/aab-61-441-2018-supplement. [file aab-61-441-supplement.pdf]

Supplement of Arch. Anim. Breed., 61, 441–449, 2018
https://doi.org/10.5194/aab-61-441-2018-supplement
© Author(s) 2018. This work is distributed under
the Creative Commons Attribution 4.0 License.

Open Access
Archives Animal Breeding

Supplement of

Polymorphisms in pattern recognition receptor genes of indigenous and White Leghorn breeds of chicken

Santosh Haunshi et al.

Correspondence to: Santosh Haunshi (santosh575g@gmail.com)

The copyright of individual parts of the supplement might differ from the CC BY 4.0 License.

S1 Table. Sequences of primers used to amplify fragments of PRR and β-actin genes along with their Ta and size of fragments

Gene	Forward (5' to 3')	Reverse (5' to 3')	Ta (^oC)	Size (bp)	Location in mRNA / CDS (bp)
TLR3	GGATGCTCTATTCCTTGCTGGA	TACAACACAAGCTCACGAAGG	62	533	204-736
	AGAACCTTCGTGAGCTTGTG	ATGTGTAATGCTGGACAATCC	60	467	712-1178
	GGATTGTCCAGCATTACACAT	ACAGTCAGATTCCGCAGTGG	60	545	1158-1702
	TTCCTCCTTGGGACAACTGA	ATGCTTGAGTGTCGTTTAGCC	60	719	1454-2172
	GCCAGCGACTTTATTTGATGAC	CCGGTAGTCTGTCAAGCTCT	60	452	1976-2427
	CGGGAGTGTTTGATTATGACG	TAGCAACCTTCAGTGACTTATTCCA	60	561	2425-2985
TLR1LA	CAGAGCTTGACTTTAGTGCCTTC	ATTCAGTCAGCTCCACCACT	62	621	498-1118
	TCCCTCCCCTTCTTTAGAGC	TGGCAAGCACTCAAACACA	64	673	940-1612
	CAGTGGGCTGATTCTCTGG	CGCGTTCGCTGTAGGAAATG	62	679	1538-2216
	ACCTGGATGTGCCGTGGT	CACTTTGCTCCTCATCTGTTTC	62	573	2085-2687
	GCAAAGAGAACGTACCTGGAGTG	CCAACTTCCAGCTTTTACCC	62	716	2534-3249
MDA5 (IFIH1)	GCGGGATTCATGTTAAGTGCCC	TCTCTGTTCCCACGACTCTCA	65	620	2-621
	GAACAGAGACGGTGCAAGGG	ATTCTCCAGGATCTGTGCTGT	64	766	613-1378
	GAGCTGAAAATCTCGTTTCCTGA	TCCCTAATCACCCACTGTTCA	66	569	1304-1872
	TGAACAGTGGGTGATTAGGGAA	CTCCTGAGCCACTTGAAGCC	66	731	1852-2582
LGP2	GCACTTATGCACTTGTGGCTT	CTCTACAGGACACAGGAGCTT	65	669	2547-3215
	AGCTCCACGGGTACCAACT	TGCTCCTGTGCTGATGCGAT	66	604	5-608
	GTGATCGCATCAGCACAGGA	CCGTAGCGGAACAGTGTGAT	65	698	586-1283
	CTTCACCAAGACCCGGCAG	GGCCACATTGCAGTTGACAC	64	571	1110-1680
	AGTACCGCCTGAAGATCGTG	ACAGGGACTCGTCCTGAGTG	65	480	1544-2023
NOD1	AGGCCACCTTTGTGCTAACT	TTGACACACTTCACCCAGGC	66	547	130-676
	GAGGGAAGAGATGCTGCTTGA	ATCTCAGTTCCTGTCCGTGC	66	567	586-1152
	CCCTCTGGTGCTGTTGGTAAG	AAGCGGTGAAAAAGGACTGAAG	66	650	1066-1715
	GTTGGGTTTTCTCAGGACAG	GTTATCCAAATCCAGAGCCAAG	58	637	1618-2254
	GTTTGGATGCTGAGGTGTCT	CACTTCCCTCCTTCGCTAGTTAT	66	452	2075-2526
	AACTAGCGAAGGAGGGAAGTG	GCAAATGATCCGTTCTTCAC	64	469	2506-2974
β-actin	TGCTGTGTTCCCATCTATCG	TTGGTGACAATACCGTGTTCA	57	150	-

Ta: Annealing temperature
